# Correlates of Burden Among Caregivers of Latinos Living With Parkinson’s Disease

**DOI:** 10.1155/padi/7714405

**Published:** 2026-09-21

**Authors:** Nicholas Renteria, Melissa N. Pacheco, Alejandra Pawlak, Gwenevere Zuniga, Maria M. Santos, Jacob D. Jones

**Affiliations:** ^1^ Department of Psychology, California State University San Bernardino, San Bernardino, California, USA, csusb.edu; ^2^ Center on Aging, California State University San Bernardino, San Bernardino, California, USA, csusb.edu

**Keywords:** caregiver burden, caregiving, Latinos, Parkinson’s disease

## Abstract

**Introduction:**

Parkinson’s disease (PD) often leads to significant motor and nonmotor impairments, including immobility, dementia, and depression. These challenges can require the involvement of caregivers to manage the needs of individuals with PD. However, the demands placed on caregivers can lead to neglect of their own well‐being, resulting in psychological and physical strain, also known as caregiver burden. Therefore, the present study explores the experiences of caregivers of Latinos with PD and examines the association between caregiver burden, unmet emotional needs, coping strategies, and caregiver‐perceived motor experiences of daily living in the individual living with PD.

**Methods:**

Caregivers (*N* = 56) of Latinos living with PD completed validated measures, including the Zarit Burden Interview (ZBI) to measure caregiver burden, the Movement Disorder Society Unified PD Rating Scale Part II (MDS‐UPDRS Part II) to measure caregiver‐perceived motor experiences of daily living, the Brief Coping Orientation to Problem Experienced (Brief‐COPE) to assess caregiver’s coping strategies, and the Support Person’s Unmet Needs Survey (SPUNS) short form to assess unmet caregiver needs. Analyses are cross‐sectional and correlational.

**Results:**

Results found that greater caregiver burden was most strongly associated with more unmet emotional needs, followed by greater utilization of problem‐focused coping strategies and greater caregiver‐perceived functional motor impact. Caregiver burden was not correlated with care recipient’s age.

**Conclusion:**

Findings suggest that caregivers of Latinos living with PD commonly experience mild‐to‐moderate amounts of caregiver burden, which is associated with a combination of caregiver‐centric (e.g., coping strategies and unmet needs) and patient‐centric (e.g., caregiver‐perceived motor experiences of daily living) variables.

## 1. Introduction

Parkinson’s disease (PD) is a chronic neurological disorder that affects millions worldwide and is characterized by a range of motor and nonmotor symptoms. Motor symptoms include uncontrollable tremors, rigidity, slowness of movement, gait/balance difficulties, and slurred speech. Nonmotor symptoms can involve autonomic nervous system dysfunction (e.g., dizziness, sexual dysfunction, and constipation), as well as cognitive and psychological challenges (e.g., dementia, apathy, and brain fog). As these symptoms progress, individuals with PD may become unable to live independently and require assistance with activities of daily living (e.g., bathing, eating, driving, and dressing).

As a result, caregivers, typically informal or nonpaid individuals such as spouses/partners or adult children, play a central role in supporting the well‐being of individuals with PD [1]. While caregivers are essential to the care process, their needs often go unrecognized. Caregivers may experience significant emotional, physical, and financial stress, which can negatively affect their health and well‐being [2]. Caregiver burden refers to the perceived adverse impact of caregiving on a person’s emotional, social, financial, physical, and spiritual functioning [3].

Cultural norms can shape caregiving roles and expectations. In Latino families, caregiving is often viewed as a familial responsibility, which may elevate risk for caregiver burden [4, 5]. Engaging in self‐care or health coping strategies may be seen as selfish, often leading to higher levels of caregiver burden [6]. Latino caregivers are more likely to prefer to care for the family member at home as opposed to placing them in long‐term assisted living facilities even in advanced stages of the disease, meaning they may care for the family member for extended periods of time [4, 5]. A study of patients with Alzheimer’s disease and their families found that Latino families may be more dismissive of Alzheimer’s disease symptoms, considering them a normal sign of aging as compared to other ethnicities [6]. This perception may contribute to the caregiver’s belief that they can provide care for their loved ones despite a lack of resources and limited access to healthcare as historically seen in Latino populations [7].

Several interconnected cultural values shape how caregiving is understood and practiced in Latino families. Familismo captures the deep sense of family unity and mutual obligation where caring for an ill relative is understood as a shared duty rather than an individual choice [8]. For many Latinos in the United States, taking on a caregiving role is simply part of what family members do, with this expectation reinforced across generations even as competing work demands create new pressures [9]. Marianismo further concentrates this responsibility on women, reflecting a cultural ideal of feminine devotion and selflessness [8]. Roughly 74% of Latino caregivers are women, and 40% of those who are employed have had to reduce hours, take leave, or exit the workforce entirely to fulfill caregiving duties [10]. Complementing these values, respeto and dignidad, which reflect deep reverence for elders and the preservation of their role and autonomy within the family, further reinforce the expectation that care be provided by family members rather than outside services [6, 8]. Together, when caregiving becomes a moral and cultural requirement, the pressure to meet that expectation without seeking outside help may intensify the strain experienced over the course of a progressive illness such as PD.

Regarding studies of caregivers of individuals with PD, over 40% of caregivers report that their physical health declined as a result of caregiving, two‐thirds report strained personal relationships, and approximately half report elevated symptoms of depression [11]. Research has explored coping strategies as potential moderators of caregiver burden. For instance, one study found that caregivers who experience higher caregiver burden tend to use a problem‐focused coping strategy, independent of other coping styles, relationship quality, and severity of the patient’s PD diagnosis [12]. Problem‐focused coping strategies are known as thoughts or behaviors that are specifically focused on the problem and creating solutions and considering different alternatives of their costs and benefits, ultimately focusing on solving problems rather than managing emotional reactions to the problems [13].

To our knowledge, no prior studies have specifically investigated the correlates of burden among caregivers of Latinos living with PD. The present study aims to explore the experiences of caregivers of Latinos with PD and examine the association between caregiver burden, unmet emotional needs, coping strategies, and caregiver‐perceived motor experiences of daily living in the individual living with PD.

## 2. Methods

### 2.1. Participants

Data were obtained from the online FoxInsight (FI) study, a national online study of people with and without PD. For up‐to‐date information on the study, visit https://foxinsight-info.michaeljfox.org/insight/explore/insight.jsp. Consent was obtained from all participants prior to study activities. The use of deidentified data from the FoxInsight repository was determined to be exempt by the California State University San Bernardino Institutional Review Board (IRB) (#2021246).

Data were downloaded from the FI repository on November 15, 2024. We identified an initial pool of 4014 participants who completed the Care Partner Experiences survey. We then sequentially restricted the sample to participants who (1) did not report a diagnosis of PD (*n* = 3855); (2) identified as a care partner for someone with PD (*n* = 2100); and (3) reported that the ethnicity of the patient/care recipient with PD was Hispanic, Latino/a/x, or of Spanish origin (*n* = 56). This resulted in a sample size of 56 caregivers of Latinos living with PD for all analyses. Notably, eligibility was based on the ethnicity of the patient/care recipient rather than the caregiver, meaning not all caregivers necessarily identified as Latino themselves. Findings should therefore be interpreted as reflecting caregivers of Latino patients with PD rather than Latino caregivers specifically.

### 2.2. Measures

Caregiver burden was assessed from the Zarit Burden Interview (ZBI‐22) [14]. Participants were instructed to indicate how often they experienced feelings associated with caregiver burden when providing care. They answered the questions using a 5‐point Likert scale, with higher scores indicating greater burden. Scores > 20 indicate mildly elevated burden, and scores > 40 indicate moderate to severe amounts of burden.

Caregiver‐perceived motor experiences of daily living in the person with PD were assessed using the Movement Disorder Society Unified PD Rating Scale Part II (MDS‐UPDRS Part II) [15]. Caregivers rated the average severity of various symptoms/functions experienced by the Latino individual living with PD over the past week. Higher scores indicated greater caregiver‐perceived functional motor impact in daily living. Because the MDS‐UPDRS Part II ratings were completed by caregivers in the present study, scores were interpreted as caregiver‐perceived motor experiences of daily living rather than clinician‐rated motor severity.

Care partner coping styles were assessed from the Brief Coping Orientation to Problems Experienced (Brief‐COPE) [16]. Participants used a four‐point Likert scale to indicate how often they utilize various problem‐focused, emotion‐focused, or avoidant coping strategies. Higher scores indicated whether the respondent used the specific coping strategy more frequently in responding to caregiver stress, while lower scores indicated that the coping strategy was less frequently used.

Unmet needs of the caregiver were assessed from the Support Person’s Unmet Needs Survey (SPUNS), short form [17]. The SPUNS was selected because it provides a multidimensional assessment of caregiver unmet needs across informational, emotional, financial, and healthcare‐related domains, consistent with the exploratory aim of examining a broad range of factors potentially associated with caregiver burden. Although the SPUNS was originally developed and validated among support persons of individuals with cancer rather than PD, its assessment of general supportive care needs was considered applicable to the present caregiving context. Participants answered self‐report items of perceived unmet needs from the following five domains: information about the condition, worry about the future, work and financial needs, access to medical care, and personal and emotional needs of the caregiver. All questions asked respondents to respond in terms of the last month. Higher scores indicated the caregiver experienced/perceived greater unmet needs in that specific domain.

### 2.3. Analyses

Pearson’s correlations examined correlations of caregiver burden with the following: caregiver‐perceived motor experiences of daily living of the Latino individual living with PD, caregiver coping styles (problem‐focused, emotion‐focused, or avoidant coping strategies), and unmet needs (information about the condition, worry about the future, work and financial needs, access to medical care, and personal and emotional needs).

In order to examine which variables were uniquely associated with caregiver burden, a stepwise regression was conducted with caregiver burden entered as the dependent variable. Independent variables included variables that were significantly correlated with caregiver burden from the Pearson’s correlations described above. In addition, demographic variables (caregiver/patient age and caregiver/patient gender) were entered as independent variables. All independent variables were entered in “stepwise” fashion, meaning that variables were entered incrementally and only if they significantly improved the model. Given the limited prior research examining correlates of caregiver burden specifically among caregivers of Latino individuals living with PD, there was insufficient empirical evidence to specify an a priori theoretical ordering of predictors for hierarchical regression. Therefore, the stepwise approach was selected to support the exploratory aim of identifying which candidate caregiver‐ and patient‐related factors demonstrated unique associations with caregiver burden. Given the data‐driven nature of variable selection and the relatively small sample, results from this analysis are interpreted as hypothesis‐generating rather than confirmatory.

## 3. Results

### 3.1. Demographic and Clinical Characteristics

Demographic and clinical characteristics of the caregiver and patient/care recipient are included in Table 1. 14.3% (*n* = 8) and 21.4% (*n* = 12) of caregivers reported mild and moderate/severe amounts of caregiver burden, respectively. Cronbach’s alpha was acceptable for all scales and subscales, ranging from 0.94 (ZBI‐22) to 0.71 (work and financial subscale of SPUNS).

**TABLE 1:** Demographic and clinical characteristics (*N* = 56).

	M	SD	Range
Caregiver characteristics			
Age	60.4	12.0	32–80
% Male	24%	—	—
% Hispanic/Latino/Spanish origin	42%	—	—
% Unpaid caregiver	100%	—	—
% Receiving assistance from another caregiver	37.5%	—	—
Relationship to patient/caregiver	—	—	—
Spouse	64.3%	—	—
Parent	19.6%	—	—
Other family member	10.7%	—	—
Other nonfamily member	5.4%	—	—
Zarit caregiver burden	23.5	17.3	2–62
Problem‐focused coping	14.9	6.1	8–28
Emotion‐focused coping	22.5	7.1	12–40
Avoidant coping	10.9	3.8	9–26
Unmet needs–information	4.5	4.2	0–13
Unmet needs–future worry	3.1	2.3	0–8
Unmet needs–work and finance	1.8	2.9	0–10
Unmet needs–access to care	4.7	4.4	0–17
Unmet needs–personal and emotional	14.4	10.8	0–39
Patient/care recipient’s characteristics			
Age	69.6	10.6	34–87
% Male	32%	—	—
% Hispanic/Latino/Spanish origin	100%	—	—
MDS‐UPDRS II^∗^	20.1	11.8	0–43
Years diagnosed with PD	10.5	5.5	1–24

*Note:* MDS‐UPDRS II = Movement Disorder Society Unified Parkinson’s Disease Rating Scale Part II.

^∗^Completed by the caregiver.

### 3.2. Regression of Caregiver Burden

Regression diagnostics indicated no substantial violations of model assumptions. Visual inspection of residual plots supported the assumptions of linearity, homoscedasticity, and approximate normality of residuals. Standardized residuals ranged from −2.68 to 2.14, with no observations exceeding ±3, and Cook’s distance values were below 1.0 (maximum = 0.63), indicating no highly influential observations.

Stepwise regression analyses examined the unique associations between caregiver burden and caregiver‐perceived motor experiences of daily living, various unmet needs, and coping styles (Table 2). The final model was significant (*F* (3, 52) = 37.6, *p* < 0.001; adjusted *R* ^2^ = 0.701). There was no indication of significant multicollinearity among predictors (tolerance > 0.5 and variance inflation factor < 2). Greater caregiver burden was uniquely associated with greater caregiver‐perceived functional motor impact, greater unmet emotional needs, and utilizing problem‐focused coping styles more often.

**TABLE 2:** Regression of caregiver burden (*N* = 56).

Parameter	Unstandardized estimate	Standard error	Standardized estimate	*p* value
Caregiver‐perceived functional motor impact	0.294	0.137	0.194	**0.037**
Unmet emotional needs	0.871	0.186	0.526	**< 0.001**
Problem‐focused coping	0.796	0.323	0.273	**0.018**
Model summary	*R* ^2^ = 0.720	Adjusted *R* ^2^ = 0.701	Tolerance ranges = 0.5–0.7	VIF ranges = 1.3–1.9

*Note:* Dependent variable = caregiver burden. Bold values represent *p* < 0.05.

Abbreviation: VIF, variance inflation factor.

### 3.3. Correlates of Caregiver Burden

Pearson’s correlations are displayed in Table 3. Greater caregiver burden showed a significant positive association with all domains/subscores of caregiver‐perceived motor experiences of daily living, unmet needs, and coping styles (Pearson’s *r* ranged from 0.357 to 0.798; all *p* values < 0.031), but was not significantly associated with patient age. Greater caregiver burden showed the strongest correlations with greater unmet emotional needs (Pearson’s *r* = 0.798, *p* < 0.001), reporting problem‐focused coping styles (Pearson’s *r* = 0.714, *p* < 0.001) and unmet informational needs (Pearson’s *r* = 0.670, *p* < 0.001). Caregiver burden was not correlated with care recipient’s age (Pearson’s *r* = 0.020, *p* = 0.447).

**TABLE 3:** Correlates of caregiver burden.

	Caregiver burden	
	Pearson’s correlation	*p* value
Caregiver‐perceived functional motor impact	0.539	**< 0.001**
Unmet needs–information	0.670	**< 0.001**
Unmet needs–future worry	0.414	**0.002**
Unmet needs–work and finance	0.563	**< 0.001**
Unmet needs–access to care	0.357	**0.006**
Unmet needs–emotional	0.798	**< 0.001**
Coping–problem focus	0.714	**< 0.001**
Coping–emotional focus	0.621	**< 0.001**
Coping–avoidant	0.493	**< 0.001**
Caregiver’s age	−0.272	**0.031**
Patient’s/care recipient’s age	0.020	0.447

*Note:* Bold values represent *p* < 0.05.

## 4. Discussion

### 4.1. Summary of Findings

The current study examined factors associated with caregiver burden among caregivers of Latinos living with PD, a population underrepresented in existing caregiver research. Results revealed that greater caregiver burden was positively associated with more unmet emotional needs, increased utilization of problem‐focused coping strategies, and greater caregiver‐perceived functional motor impact in daily living.

In our sample, 35.7% of caregivers reported clinically elevated levels of burden, suggesting a substantial proportion of caregivers of Latino individuals living with PD are affected. Although our study did not include a comparison group, this burden rate appears consistent with or slightly elevated compared to estimates from samples of predominantly non‐Latino individuals living with PD. Caregiver burden in PD varies widely across the literature, with ZBI scores ranging from as low as 14.4 (minimum burden) to over 47.4 (severe burden) [1]. This variability in findings likely reflects differences in the patient and caregiver population (e.g., disease stage, informal vs. formal caregivers), methodological differences in the recruitment of participants (e.g., community versus clinical sample), and structural differences in healthcare access across study settings. Prior studies have found that burden increases as the disease progresses [18], when care recipients experience depression, reduced functional independence, or poor quality of life, and among informal caregivers compared to formal ones [19]. Regardless of this variability in the literature, the relatively common presence of caregiver burden raises the concern that caregiver burden is understudied in PD, including the Latino PD population.

### 4.2. Cultural and Psychosocial Context

Notably, caregiver burden in this group was most strongly linked with psychosocial factors–particularly unmet emotional and coping strategies–underscoring the importance of examining how caregivers respond to stress within their sociocultural context. Research on caregivers of Latinos in other illness contexts, such as Alzheimer’s disease and cancer, has similarly identified high emotional strain, unmet needs, and psychological distress, often influenced by cultural expectations around family caregiving, limited access to linguistically and culturally appropriate support, and broader systemic inequities [20, 21]. Our findings mirror these patterns, highlighting unmet needs and informational gaps as key contributors to caregiver burden. The results underscore the importance of addressing cultural and structural barriers to support. Future research should develop culturally informed interventions that target emotional well‐being and access to resources for caregivers of Latinos.

### 4.3. Coping and Emotional Needs

For caregivers of Latinos with PD, addressing unmet emotional needs may require interventions that are both evidence‐based and culturally responsive. Emotion‐focused coping strategies–such as stress reduction, emotional regulation training, and acceptance‐based approaches–have been associated with improved quality of life and, in some cases, reduced rates of institutionalization in dementia caregiving contexts [22, 23]. Similarly, psychosocial interventions that incorporate stress management, emotional support groups, and caregiver education have shown promise in reducing depression and burden [24]. To be effective for Latino patient populations, these interventions should be grounded in culturally relevant values and support systems such as family values, language preferences, and cultural expectations around caregiving [22].

Although problem‐focused coping is often thought to reduce caregiver burden by promoting active problem‐solving and planning [13], our findings suggest a more complex relationship. In our sample, greater use of problem‐focused coping strategies was significantly associated with higher caregiver burden. This is consistent with findings from Zhang et al. [12], who reported that problem‐focused coping was positively associated with greater caregiver burden and remained a significant predictor of burden after accounting for PD severity, relationship quality, and caregiver gender. They proposed that in progressive conditions such as PD–where symptoms worsen over time–problem‐solving efforts such as researching the disease or implementing safety strategies may not meaningfully reduce the emotional or physical demands of caregiving. These strategies may help caregivers feel in control of day‐to‐day tasks, but they do not necessarily mitigate deeper sources of distress, such as grief or anticipatory loss. Similarly, Cooper, Katona, Orrell, and Livingston [25] found that dementia caregivers who used fewer emotion‐focused strategies combined with more problem‐focused coping reported greater anxiety 1 year later, suggesting that such strategies may be less effective in long‐term, low‐control caregiving contexts.

### 4.4. Cultural Considerations in Coping Strategies

In Latino caregiving contexts, problem‐focused coping may include strategies such as rationalization or gaining understanding of the care recipient’s condition, which are seen as culturally congruent forms of active coping. One study of Latino caregivers of individuals with Alzheimer’s disease and related dementias found that rationalization–interpreting challenging behaviors as symptoms of illness–was commonly used to assess and manage caregiving stress [26]. While this approach reflects active engagement with the problem, it may not sufficiently address the emotional impact of caregiving in progressive diseases such as PD. These culturally grounded strategies, though adaptive in intent, may fall short when the caregiving challenges are persistent and unresolvable, possibly contributing to increased caregiver burden.

Beyond rationalization, Latino patients and their caregivers may also draw on religion, spirituality, and informal family networks as additional coping resources, with research showing that Latinos tend to pray more and involve family members as helpers rather than turning to formal support services [6]. Latino caregivers are also less likely to utilize formal care services compared to their non‐Latino counterparts, which may further limit available coping resources [4]. However, despite the cultural emphasis on family involvement, many Latino caregivers find themselves as the primary or sole provider, as the assumed availability of family support does not always match reality [6]. These patterns highlight the need for culturally informed intervention that acknowledge the full range of coping strategies Latino patients and their caregivers draw upon.

## 5. Future Directions, Limitations, and Conclusions

Findings from this study suggest that caregivers of Latino individuals with PD commonly experience mild‐to‐moderate levels of burden, which appear to be shaped by both caregiver‐centric factors–such as coping strategies and unmet emotional needs–and patient‐centric functional factors as perceived by caregivers, such as motor experiences of daily living. Notably, problem‐focused coping strategies were associated with higher burden, highlighting the need to better understand how coping approaches function in the context of chronic and progressive illness. Future research should explore whether culturally tailored interventions that support emotional well‐being and flexible coping can help reduce caregiver burden in Latino families affected by PD.

Limitations are present in the study. The study used a cross‐sectional and correlational design, which prevents conclusions about causality or directionality. The sample size, *N* = 56, is relatively small. However, racial/ethnic minorities have been severely underrepresented in PD research, and concerns about statistical stability should be balanced against an ethical obligation to include underserved populations in research. We recognize that stepwise regression methods may produce unstable models and therefore interpret these findings as hypothesis‐generating rather than confirmatory. In addition, participants were drawn from FI, an online, self‐selected research registry, which may introduce selection bias related to internet access, comfort with online surveys, health literacy, and connection to PD research networks. As a result, findings may not generalize to all caregivers of Latino individuals living with PD, particularly caregivers who face greater structural barriers to research participation. Patient motor experiences of daily living were reported by caregivers rather than directly assessed by clinicians; therefore, MDS‐UPDRS Part II scores should be interpreted as caregiver‐perceived motor experiences of daily living rather than clinician‐rated motor severity and may partly reflect caregiver perception or caregiving‐related stress. In addition, the SPUNS measure was developed and validated in a non‐PD medical population, which may limit the extent to which its items fully capture the unique unmet needs of caregivers of Latino individuals living with PD.

Although gender was not a primary focus of the present study, it is an important consideration for future research. While the following concepts were not measured in the current study, cultural values such as marianismo and familismo place caregiving disproportionately on women, framing it as a moral duty and natural extension of feminine identity [6, 8]. These gendered burdens may shape the caregiving experience in meaningful ways that the present study was not designed to capture. Future research should examine whether gender moderates the relationship between caregiver burden and its correlates in caregivers of Latino individuals living with PD and the role of concepts such as familismo, marianismo, respeto, and dignidad.

## Funding

This study was funded by the National Institute of Neurological Disorders and Stroke (SC3NS124906), the National Institute of General Medical Sciences (T34GM136467), and the National Institute of Mental Health (K23MH119313).

## Conflicts of Interest

The authors declare no conflicts of interest.

## Data Availability

The data that support the findings of this study are available from the Fox Insight study repository. Access to the data is subject to approval and data use agreements through Fox Insight (https://foxinsight-info.michaeljfox.org/insight/explore/insight.jsp).
